# Mirtazapine-induced psychosis on a young patient with severe malnutrition

**DOI:** 10.1192/j.eurpsy.2023.2155

**Published:** 2023-07-19

**Authors:** R. T. E. Sollano, A. K. L. Enriquez

**Affiliations:** 1 Psychiatry, The Medical City - Ortigas, Pasig, Philippines

## Abstract

**Introduction:**

Mirtazapine is an antidepressant commonly prescribed to patients with depression and problems with weight and sleep. Case reports on Mirtazapine-induced psychosis either on initiation or increase in dosage in elderly patients and those with renal and liver impairment are found in the literature.

**Objectives:**

To present a case of Mirtazapine-induced psychosis in a patient with severe malnutrition, and with no history of psychosis and despite on sedating antipsychotic.

**Methods:**

This is a case report.

**Results:**

Ms. NC, a 40-year-old female with major depressive disorder, anorexia nervosa, stimulant use disorder, and sedative, anxiolytic, hypnotic use disorder with no history of psychosis even when intoxicated or during withdrawal, was admitted for involuntary inpatient psychiatric care for detoxification and management of severe malnutrition. Ms. NC has always been conscious with her weight growing up but it was only during the COVID-19 pandemic that excessive preoccupation with weight and symptoms of clinical depression were noted. Ms. NC restricted her diet and engaged in excessive exercise resulting to BMI of 16.1. She started use cocaine and diazepam daily to address the weight and mood, and sleep and anxiety, respectively. Due to a suicidal attempt, consult was done with a psychiatrist, and patient was eventually maintained on Mirtazapine 30mg and Gabapentin 100mg which addressed the mood and sleep. Despite improvement in mood and decrease in use of cocaine and diazepam, patient started to use methamphetamine around once a week. Despite with euthymic mood, preoccupation with weight resurfaced. After a few months, she restricted her food intake to only four times a week with no binge-eating or purging resulting to BMI to 13.8. Upon admission, Mirtazapine 30mg was continued and Gabapentin was increased to 300mg. Special care in her food intake was done to prevent refeeding syndrome. Benzodiazepine withdrawals symptoms were minimal. She has normal values for electrolytes, liver function tests and creatinine. On the first days of admission, she was noted to be irritable and was mostly asleep. On the fifth hospital day, she started to have difficulty sleeping and was placed on Olanzapine up to 10mg and Gabapentin 600mg but no improvement in sleep. On the tenth hospital day, Mirtazapine was increased to 45mg and later in the night, had visual and auditory hallucinations and paranoia. Upon discontinuation of Mirtazapine and initiation with Clozapine up to 75mg, the psychosis resolved after five days.

**Conclusions:**

Mirtazapine-induced psychosis may be seen in patients with severe malnutrition. Despite its advantages in terms of weight gain and sleep, psychiatrists should be wary of this possible side effect when initiating or increasing Mirtazapine for patients with severe malnutrition.

**Disclosure of Interest:**

None Declared

